# Natural disasters in the United States: Hurricane risk, hospital closures, and healthcare finance

**DOI:** 10.63564/jha.v14n2p16

**Published:** 2025-09-03

**Authors:** George Audi, Hanadi Hamadi, Margaret Capen, Rima Tawk, Willie Williams

**Affiliations:** 1 School of Allied Health Sciences, Florida A&M University, Tallahassee, Florida, United States; 2 Brooks College of Health, University of North Florida, Jacksonville, Florida, United States; 3 College of Business, East Carolina University, Greenville, North Carolina, United States; 4 School of Public Health, Florida A&M University, Tallahassee, Florida, United States

**Keywords:** Cost-to-charge ratio, Disaster preparedness, Hospital finance, Hurricane risk, Rural hospitals

## Abstract

**Background:**

Global climate change has increased the likelihood of natural disasters, including hurricanes, floods, wildfires, tornadoes, and earthquakes; this increased risk presents acute socioecological disturbances that generate cascading impacts across healthcare systems, social structures, and economic frameworks. Forty-three percent of Atlantic hurricanes that make U.S. landfall hit the southeastern United States, and their increasing intensity threatens the healthcare infrastructure. Hospital cost-to-charge ratios (CCRs) vary between rural and urban facilities, but hurricane risk impacts on hospital financial performance remain poorly understood.

**Objective:**

To examine relationships among hurricane risk, geographic location, and hospital CCRs among southeastern hospitals.

**Methods:**

A cross-sectional analysis was used to merge 2021 CMS Cost Report data with 2023 FEMA National Risk Index data for 1,030 hospitals across eight southeastern states. All hospitals within this region were included except for federally funded hospitals due to their unique funding model. Each hospital self-reports its categorization of urban or rural on the CMS Cost Report. Multivariate regression was used to examine associations among log-transformed CCR and hurricane risk percentile, rural/urban location, and hospital quick ratio.

**Results:**

Among 1,030 hospitals analyzed, 52% were rural and 48% urban. The regression model explained 24.7% of CCR variation (adjusted *R*^2^ = 0.2465, *F* = 85.18, *p* < .0001). All predictors were statistically significant (*p* < .0001). Counter to expectations, each 1-point increase in hurricane risk percentile was associated with a 0.1% decrease in CCR, indicating improved cost efficiency in higher-risk areas. LOGCCR = - .75714 - .00840 (NAPCT) - .26551 (RURAL) + .01491 (QUICK) - .00011 (QUICK^2^). Rural hospitals as indicated by the CMS Cost Report demonstrated 26.5% lower CCR compared to urban hospitals. Hospital quick ratio showed a curvilinear relationship with CCR; at the mean quick ratio (3.819), each 1-unit increase was associated with a 1.4% increase in CCR. No significant multicollinearity was detected among predictor variables.

**Conclusions:**

Hurricane risk is paradoxically associated with lower hospital CCR, suggesting complex financial adaptations in high-risk areas. Rural hospitals maintain more favorable cost structures than urban facilities, and policymakers should consider these geographic variations in disaster preparedness strategies.

## 1. Introduction

### 1.1 Current trends

Natural disasters, including hurricanes, floods, wildfires, tornadoes, and earthquakes, represent acute socioecological disturbances that generate cascading impacts across healthcare systems, social structures, and economic frameworks. Contemporary climate science demonstrates that these catastrophic events are becoming increasingly frequent and intense due to climate change and environmental degradation, particularly in the United States.^[1,2]^ The geographical and climatological diversity of the United States creates a complex landscape of disaster vulnerability that varies significantly across regions, with the southeastern states experiencing disproportionate exposure to hurricane events.^[3]^ According to the National Oceanic and Atmospheric Administration, the United States now experiences an average of 15 major disasters annually, with associated economic costs exceeding $1.8 trillion between 2010 and 2023.^[2,4]^ Hurricane events comprise approximately 60% of these costs. However, despite the economic magnitude of these impacts, theoretical frameworks for understanding how disaster vulnerability intersects with healthcare infrastructure resilience remain unknown, particularly concerning rural-urban disparities in healthcare finance.^[5]^

The southeastern states, particularly within the Gulf Coast region, have been susceptible to more than 40% of hurricanes that land in the United States.^[6,7]^ Therefore, the southeastern United States represents a region of particular concern for hurricane vulnerability assessment. Both frequency and intensity metrics demonstrate concerning increasing trends, with the 2020–2023 Atlantic hurricane seasons averaging 19.5 named storms per year compared to the historical average of 14.4 storms annually.^[2]^ More critically, rapid intensification events, in which storm wind speeds increase by at least 35 mph within 24 hours, have increased by 38% since 1979, according to recent satellite data analysis.^[8]^ This intensification creates challenges for hospitals’ disaster planning by compressing response timeframes. Regional analysis reveals that 43% of Atlantic hurricanes making landfall in the United States between 2000–2023 affected southeastern states, with Florida, North Carolina, and Louisiana experiencing the highest frequency of direct impacts.^[7]^

Hurricane damages are not distributed uniformly among these states; they follow demographic and infrastructure vulnerability patterns that often correspond with healthcare access challenges.^[8]^ Previous research has been focused extensively on immediate physical damages to healthcare infrastructure during hurricane events.^[9,10]^ However, methodological limitations have constrained the understanding of longer-term financial impacts. Most researchers employ case study approaches examining individual facilities or events (e.g., Hurricane Katrina, Hurricane Ian) rather than systematic longitudinal analyses.^[11]^ Notwithstanding, few studies have involved control comparisons to isolate hurricane effects from other healthcare finance trends, creating challenges for causal inference.

### 1.2 Financial impact

The significant financial impact of yearly major disasters such as Hurricane Katrina (2005), Hurricane Irma (2017), and Hurricane Ian (2022) underscore the short- and long-term effects on health and infrastructure and the significant losses due to floods, storm surges, and wind effects.^[10]^ Additional compounding factors, such as disruption in the transportation of resources, supplies, and people with limited access to healthcare or low-income status, are the most vulnerable in such events.^[9,12]^

The financial vulnerability is particularly acute for hospitals serving low-income populations, as these facilities often operate with thin margins and limited financial reserves. Post-hurricane periods frequently involve increased demand for emergency services and reduced revenue due to postponed elective procedures and patient displacement. Supply chain disruptions can increase operational costs by 15%-30% in the weeks following major hurricanes, and staffing shortages due to evacuation and infrastructure damage further strain hospital budgets.^[12]^

Beyond immediate impacts, hurricanes create long-term financial pressures through infrastructure rebuilding costs, increased insurance premiums, and the need for enhanced disaster preparedness investments. Rural hospitals face disproportionate challenges, as their smaller patient bases and limited financial resources make recovery more difficult. Some facilities never fully recover financially, contributing to the pattern of rural hospital closures observed in hurricane-prone regions. Understanding these financial dynamics is crucial for developing sustainable healthcare delivery models that can withstand increasingly frequent and severe weather events while maintaining access to essential medical services for vulnerable populations.

### 1.3 Effect on healthcare industry

Natural disasters affected by climate change, particularly hurricanes, affect healthcare systems, causing a byproduct of unplanned interruptions in treatment processes, increased use of emergency services, and the need for prompt medical response.^[2]^ The strain on hospital care delivery systems is evident across all four domains: infrastructure, resources, patients, and staff. In addition to providing acute care, healthcare providers must navigate patients’ mental health status, chronic diseases, physical damage to hospital structure, short supplies of medicines, power disruptions, high utilization of health facilities, and personnel exhaustion. Structural preparedness is a key to contingency hospital adaptation in natural disasters. Specific building codes enforced on existing health facilities ensure that structures used as hospitals can withstand earthquakes, floods, or any other likely disasters.^[13]^

The Centers for Disease Control and Prevention emphasizes the importance of public health coordination at the organizational and community levels to promote preparedness for natural disasters. As natural disasters become more frequent and intense, healthcare systems must continually explore and implement diverse strategies. Structural preparedness, adequate funding, trained personnel, and community engagement in disaster response will help mitigate the disruption of patient care services.

### 1.4 Effect on hospitals

The U.S. hospital landscape comprises over 6,000 hospitals that vary significantly in size, service offerings, and financial structures.^[14]^ Understanding the preexisting structural differences between rural and urban hospitals is essential for contextualizing differential hurricane impacts. Urban hospitals typically feature larger bed capacity (average of 270 beds versus 25 beds in rural facilities), higher staffing ratios, more specialized technologies, and greater service diversification.^[15]^ These structural advantages create operational redundancies that can enhance disaster resilience. For example, urban facilities can often reallocate staff across departments during emergency events or transfer patients within networked systems.^[16]^ In contrast, rural facilities typically operate with minimal redundancy and limited specialty coverage, creating points of increased vulnerability during disruptions.^[17]^

Urban and rural hospitals differ in various ways, primarily due to their geographical location and the population they serve. Urban hospitals tend to be supported by more capital and higher insured populations that contribute to better provisions of facilities, staff, and better-specialized technologies.^[15,16]^ Rural hospitals, on the other hand, are typically small organizations that may not have the same financial resources as large, multi-site, and multi-hospital systems; they are focused on meeting the needs of people living in less populated and remote regions. Rural hospitals are also less equipped and generally have few beds and fewer services than larger city hospitals. They are usually more customer-oriented because of the closer proximity to service end users. However, they may incur problems delivering the same service diversification and technological sophistication.^[18]^ Rural hospitals also face financial challenges and a higher risk of closure due to their smaller patient base and higher proportion of uninsured or underinsured patients, making it hard to fund themselves.^[11]^ One of the main issues is the shortage of healthcare professionals, including doctors and specialists, causing residents to travel for services.^[17]^

Hospital closures in rural and urban areas have become a significant issue in the healthcare system, leading to widespread disruption of care for patients.^[19]^ In rural hospitals, factors contributing to closures include systemic and financial vulnerability, limited patient base, inadequate staffing, and challenges in containing specialized clinical services or technology.^[20]^ The dynamics involving urban hospitals differ slightly, mainly resulting from mergers, transformation of the healthcare system, or corporate decisions to transition to centralized services and downsize.^[21]^ Restructuring to increase organizational efficiency results a decreased number of hospital beds available, increased service waiting time, and less provision of specialty services. Closures ultimately disproportionately impact low-income residents already experiencing barriers to accessing healthcare services and adequate primary care physicians.^[20]^ Hospital closures strain nearby healthcare facilities, which can cause collapse due to the congestion of unreimbursed care from patients.^[22,23]^ By increasing funding support at the policy level, measures can be taken to promote sustainable healthcare delivery models.^[24]^

During hurricanes, hospitals see an influx of patients that strains resources, compromises care quality, and delays providing care to patients with chronic illness or injury.^[25]^ Under normal circumstances, digital health technology can assist with solving these problems because it facilitates efficient resource centralization and coordination.^[26]^ Additionally, high burnout among healthcare workers caused by physically and emotionally challenging expectations influences the stability of a hospital’s workforce during natural disasters. For example, nephrology staff were distressed due to long working hours and patients’ escalating needs.^[27]^ The synergism of fatigued healthcare workers, interrupted communication, and supply chain breakdowns can limit the effectiveness of patient care services.

Utilizing past disaster management experiences to inform the current preparation of current healthcare systems can save people and money and aid in delivering quality healthcare services after a disaster. Unfortunately, recurring errors in planning and resource allocation amongst hospitals highlight the lack of insight gained from previous disasters,^[28]^ considering the effects of hurricanes on numerous dimensions: structures, patients, resources, communication, and healthcare workers. Disaster preparedness must be used to expand resources in areas of surge capacity, innovative digital communication capabilities, and staff support to cover such dimensions affected by natural disasters like hurricanes.

### 1.5 Hurricane effect on hospital CCR

Understanding how hurricane vulnerability affects hospital financial viability requires examining the metrics and mechanisms through which hospital financial performance is measured and managed. The cost-to-charge ratio (CCR) represents a critical metric for assessing hospital financial efficiency. It measures what hospitals spend on delivering healthcare relative to what they charge patients. This ratio provides critical insights into operational efficiency, pricing strategies, and financial sustainability. A higher CCR, especially in institutions serving underfunded institutions, means more financial pressure and inefficiency, whereas a lower CCR can lead to cost control and utilization, thus supporting financial stability.^[29,30]^ Ultimately, this tool, which connects financial health to outcomes and efficiency, assists hospitals in aligning their cost models with strategic pricing to drive profitability and resilience.^[31]^ CCR is calculated by dividing total costs by total charges, with lower ratios indicating greater markup between costs and charges. A CCR serves as both an indicator and determinant of hospital financial health. A recent study demonstrated through multivariate analysis of 2,456 US hospitals that CCR correlates significantly with operating margin (*r* = 0.67, *p* < .001), days cash on hand (*r* = 0.54, *p* < .001), and bond ratings (*r* = 0.48, *p* < .01). These relationships remained significant after controlling for hospital size, case mix index, and geographic location.

Rural–urban disparities in CCR have been documented in several studies, though methodological limitations constrain generalizability.^[29]^ One study found that rural hospitals had CCR values averaging 0.14 points higher than urban facilities, suggesting thinner financial margins between costs and charges. This disparity persisted after controlling for payor mix, case complexity, and organizational characteristics.^[32]^ However, this analysis did not incorporate hurricane vulnerability as a potential moderating variable. Limited research has directly examined how hurricane events affect hospital financial metrics, with even fewer studies investigating CCR impacts. Therefore, this study addresses a critical knowledge gap by examining how hurricane risk interacts with the rural–urban divide to influence hospital financial sustainability.

The proactive management of CCR thus becomes a mechanism for maintaining competitiveness and driving performance improvements and patient experiences in multiple ways in the management of hospitals. A hospital with a well-functioning CCR can provide quality services at reasonable costs, bringing in more patients.^[29]^ Additionally, the role of CCR is critical in highly specialized fields such as neurosurgery, in which cost control can increase a hospital’s market profile without reducing the quality of care.^[32]^ However, solely focusing on cost effectiveness can have a direct impact on patent satisfaction, as this can decrease the quality of care provided. CCR is not only of operational and competitive value but is also vital to financial forecasting and risk management. CCR and other financial metrics indicate possible financial trouble, prompting hospitals to take preventative action to avert bankruptcy. Incorporating CCR in systems with value-based care will be critical in the future to better align clinical efficiencies within hospitals.^[31]^

## 2. Data and methodology

Data were obtained by combining two national datasets: the 2021 Centers for Medicare and Medicaid Services (CMS) Cost Report Dataset and the 2023 Federal Emergency Management Agency (FEMA) National Risk Index. The 2021 CMS Cost Report dataset captured the hospital name, its address and zip code, its locality (rural/urban) and its CCR. The national hurricane percentile risk (NAPCT) was drawn from the 2023 FEMA’s National Risk Index dataset. Data from both datasets (CMS Cost Report and FEMA’s National Risk Index) were merged by zip codes. The states that were selected for the analysis included Alabama, Florida, Georgia, Kentucky, Louisiana, Mississippi, North Carolina, and South Carolina. The combined dataset included both rural and urban hospitals. Each organization self-reported its categorization of urban or rural in the CMS Cost Report. The analysis excluded federally funded hospitals, including hospitals that are associated with Department of Defense, Public Health Service, Veterans’ Affairs (VA), PHS Indian Service, and Department of Justice hospitals, which do not report financial information due to their unique funding structure. The analysis excluded VA hospitals as they do not report financial information to CMS.

The dependent variable was each hospital’s CCR obtained from the CMS dataset. It represented a broad indication of the hospitals’ financial performance and operational effectiveness. Independent variables (NAPCT, RURAL, QUICK and QUICK^2^) were used in the regression model. NAPCT was obtained by querying the reported zip code of the hospital and ranged from 0 to 100. RURAL was coded as 1 if the hospital reported it was in a rural area or 0 if it was in an urban area. This information was self-reported by each hospital and originated in the CMS Cost Report. QUICK denoted the ratio of current assets divided by current liabilities of a given hospital and was obtained from the CMS Cost Report. The relationship between CCR and QUICK was suspected be curvilinear based on a plot between these variables. Therefore, to measure the total relationship between CCR and QUICK, an additional quadratic term (QUICK^2^) was included in the model. Because the objective of the model was to discover which independent variables were significantly related to CCR, a multivariate regression model was utilized.

## 3. Results

Based on the total number of hospitals in the dataset (1,030), 48% reported that they were in urban areas while 52% reported that they were in rural areas. A multivariate regression model was used to examine the relationship between CCR and its determinants. The initial equation (run) had a *F*-Statistics of 74.38 that was significant at a *p*-value < .0001, however, a check of the assumptions of the models indicated that the residuals violated the homoscedasticity assumption of constant variance. (CHI-SQUARE of 37.91; *p*-value = .0002). Therefore, a log transformation of CCR was utilized as the new dependent variable. The model was then re-run using the transformed variable to stabilize the variance of the residuals. The CHI-SQUARE test was 14.68 with a *p*-value of - .2594, thus, the assumption of homoscedasticity of the residuals was then met. A plot of the distribution shows slight non-normality; however, this would not have a substantive effect on the results due to the large sample size.

Referring to Table 1, the final model estimating the relationship between LOGCCR and its determinants was: LOGCCR = - .75714 - .00840 (NAPCT) - .26551 (RURAL) + .01491 (QUICK) - .00011 (QUICK^2^). A correlation matrix of these variables showed no multicollinearity.

The *F*-Test for significance of the transformed model was: 85.18 with a *p*-value < .0001 and an adjusted *R*-Square value of 0.2465, therefore after adjusting for degrees of freedom, 24.65% of the variation in CCR was explained by this model. Two-tailed hypothesis tests were conducted to check for the significance with CCR as it related to independent variables. These *t*-tests and their values were reported in Table 1. All independent variables were significant at the *p*-value of .0001.

For a quantitative independent variable such as NAPCT, its coefficient shows how a 1 unit increase in the independent variable, NAPCT, related to the change in the dependent variable LOGCCR. If we exponentiate the coefficient, we get -1.001, which indicated that for every 1-point increase in NAPCT, the CCR decreased by .0001.

The coefficient on the dummy variable (RURAL) is - .26551 and represented the average difference in CCR between Rural and Urban hospitals. If we exponentiate the coefficient on RURAL, this provided a value of -1.304, therefore rural hospitals had a CCR that was 30.4% less then urban hospitals, on average. Since the independent variable QUICK had a hypothesized curvilinear association to CCR, it was necessary to take a partial derivative to capture the total effect of the association between QUICK and CCR. This total associated value was calculated by using the mean value of QUICK from the dataset of 3.817. When we exponentiate this number, the resulting value of 1.014 infers that for every 1 unit change in QUICK, the CCR increased by 1.4%.

## 4. Discussion

Hospitals face several factors that affect their financial stability. This study examined the associations among hurricane risk, hospital setting, and CCR. Weather events, particularly hurricanes, can devastate an area by displacing a hospital’s population or disrupting its functionality or efficiencies. When taken together, a “perfect storm” could occur, resulting in a hospital going offline and then closing permanently. Hurricanes can also negatively affect the population of an area on the physical, social, and physiological levels.

Hospitals, in essence, exist to provide healthcare for the populations that they serve. Hurricane displacement could last days or even be permanent based on the severity of the hurricane and the population’s ability to obtain financial resources to resume pre-hurricane conditions. As a result, hospital administrators, policy makers and other stakeholders should increase their understanding of the adverse outcomes associated with weather-related disasters such as hurricanes and the effects that these weather-related events have on hospitals’ financial performance and security. This should include fluctuations in reimbursement rates that hospitals charge to adjust to the underlying hurricane risk. Without having this variable properly factored into their business models, hospitals could experience a catastrophic loss of revenue directly, as a hospital is taken offline due to a hurricane, their population is displaced and no longer use a certain hospital, or both. In either case, the hospital would experience lower revenue.

If this were to persist, the hospital could have prolonged financial losses that could ultimately lead to closure. While there are avenues for hospitals to receive emergency funding if they are affected by natural disasters, hospitals should not depend on this alone. Hospital stakeholders should also use information associated with hurricane risk to mobilize needed resources to mitigate the adverse health events hurricane survivors experience.

CCR is a financial metric that represents the relationship between a hospital’s costs and what it charges for services. Whereas most financial metrics are positively correlated, CCR is inversely correlated in that a CCR closer to 1 (or 100%) indicates that the hospital’s charges are closer to its actual costs, and a CCR closer to 0 indicates a higher markup on services. Therefore, a lower CCR would indicate that a hospital would be able to hedge the possibility of being offline due to an increased hurricane risk.

Extant studies have examined hospitals’ CCR regarding their organizational structure,^[33,34]^ variability in the financial strength of urban versus rural hospitals,^[5,35,36]^ the financial vulnerability and risk of closure among rural hospitals,^[19,23,37]^ and the risks that hurricanes pose for populations.^[38–41]^ To our knowledge, this is the first study to examine the impact of hurricane risk, hospital location, and quick ratio on a hospital’s CCR. Although this is a unique study, it is subject to some limitations. For instance, financial information within the CMS dataset is mostly self-reported by hospitals. The risk of hurricanes is changing, and, in many cases, other weather-related events such as tornadoes or flooding affect hospitals within southern states. Future studies should look at other types of weather-related events, which may include flooding, tornadoes, earthquakes, or a combination of each. They could also look at multi-hospital systems’ effects on hurricane preparedness and mobilization of resources and how artificial intelligence could play a role in mitigating the hazards related to weather related events.

## 5. Conclusions

Hospitals play a vital role for the populations they service. Having to navigate both the current healthcare industry and the unknown challenges that many face due to hurricane risk makes managing hospitals extremely challenging. These findings stress the importance of not only understanding the challenges that hospitals face based on urban or rural settings but other major factors, such as geographical location and the effect of coastal hurricane risk on the financial viability of hospitals. The CCR in many hospitals, along with these hospitals’ assumed hurricane risk, are important to understanding their financial viability and the effect of geographic risk.

## Figures and Tables

**Table 1.** Multivariate regression result of cost-to-charge and its determinants

Variable	Coefficients	SE	*t* value
INTERCEPT	−.75714	.05299	−14.29^*^
NAPCT	−.00840	.00074	−11.38^*^
RURAL	−.26551	.03599	−7.38^*^
QUICK	.01491	.00357	4.18^*^
QUICK^2^	−.00011	.00003	−4.21^*^

*Note. F* value = 85.18^*^; *R*-square adjusted = 0.2465; Degrees of Freedom = 1,029; Dependent Variable: LOGCCR; SE = Standard Error;

* Significant at *p* < .0001 level

## Data Availability

The datasets generated during and/or analyzed during the current study are available from the corresponding author on reasonable request.
